# Complete chloroplast sequence of common groundsel (*Senecio vulgaris* Subsp. *vulgaris*)

**DOI:** 10.1080/23802359.2019.1687352

**Published:** 2019-11-08

**Authors:** Jin-Won Kim, In-Yong Lee

**Affiliations:** Department of Agro-food Safety and Crop Protection, National Institute of Agricultural Sciences, Iseo-myeon, Wanju-gun, Jeollabuk-do, Republic of Korea

**Keywords:** Common groundsel, winter weed, exotic weed, old-man-in-the-spring

## Abstract

*Senecio vulgaris* is an exotic annual winter weed that is problematic in garlic and onion fields in Republic of Korea. The chloroplast DNA was 150,765 bp with 82,907 bp of large single-copy region, 18,214 bp of small single-copy region, and 24,822 bp of a pair of inverted repeats. A total of 104 genes were annotated including 80 protein-coding genes, 20 tRNA genes, and 4 rRNA genes. Twelve genes (*ClpP*, *ndh*B, *ycf*3, *rpl*2, *rps*12, *trnA*, *trnI*, *trnR*, *trnG*, *trnL*, and *trnV*) were multiple-copy genes. *Senecio vulgaris* was closely related to *Jacobaea vulgaris* in same subfamily resulting from phylogenetic analysis.

*Senecio vulgaris* L. (common groundsel) is a winter annual weed with 2*n* = 4*x* = 40 chromosomes (Chang and Chung 2011) and approximately 1.63 ρg 1 C^−1^ and 3.2 Gbp (Garnatje et al. 2011). *Senecio vulgaris* was divided into two subspecies: *S. vulgaris* subsp. *vulgaris* is the non-radiate form (no ray florets), and *S. vulgaris* subsp. *denticulatus* (syn. subsp. *hybernicus*) is the radiate form (ray florets). *Senecio vulgaris* subsp. *vulgaris* is distributed nationwide in Korean croplands (Kim, Kim, et al. 2018) but recently had been problematic in winter crop cultivation such as garlic and onion (Kim, Lee, et al. 2018). The objective of this study was to analyze chloroplast genome sequence of *S. vulgaris* for providing essential information to studies on genetic diversity and effective weed management.

The specimen was collected from Iseo-myeon, Wanju-gun, Jeollabuk-do, Republic of Korea (35°49′31.4″N, 127°02′41.9″E) and deposited at National Agrobiodiversity Center (K265648). Total genomic DNA was extracted from fresh leaves and genome sequencing was conducted using the Illumina MiSeq platform (Illumina Inc., San Diego, CA). High-quality paired-end reads of *ca*. 2.5 Gb were used to assemble chloroplast genome (GenBank Accession no. MH746728), as described previously (Kim et al. 2015).

Then complete chloroplast genome size is 150,765 bp. The genome consists of a large single-copy (LSC) region of 82,907 bp, small single-copy (SSC) region of 18,214 bp and a pair of inverted repeats (IRa and IRb; IRs) of 24,822 bp. A total of 104 genes were annotated in the chloroplast genome, which included 80 protein-coding genes (PCGs), 20 tRNA genes, and 4 rRNA genes. Twelve genes (*ClpP*, *ndh*B, *ycf*3, *rpl*2, *rps*12, *trnA*, *trnI*, *trnR*, *trnG*, *trnL*, and *trnV*) were multiple-copy gene. In IRs region, 8 tRNA genes, 7 protein-coding genes, and 4 rRNA genes were duplicated. Overall GC content of chloroplast genome is 37.82%.

Phylogenetic study was conducted with 11 problematic weeds in Asteraceae based on the 86 protein coding sequences by using MEGA X and outgroup was *Echinochloa oryzicola* (Poaceae) (Figure 1). As a result of 1000 bootstraps, *S. vulgaris* was closely related to *Jacobaea vulgaris* of same subfamily. The information could be essential information for genetic diversity analysis of Korea *S. vulgaris* and also be helpful to elucidate the mechanism of herbicide resistance based on DNA mutation.

**Figure 1.**
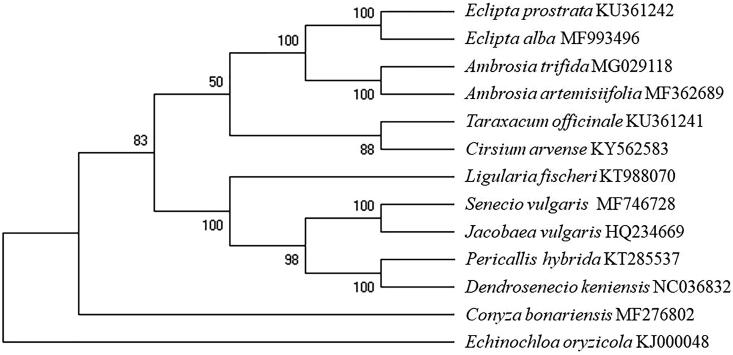
Maximum likelihood phylogenetic tree of *S. vulgaris* with 11 species in Asterceae was constructed by 86 protein coding sequences in chloroplast. Numbers on the nodes are bootstrap values from 1000 replicates. Outgroup was *Echinochloa oryzicola* (Poaceae).

## Nucleotide sequence accession numbers

The cpDNA genome sequence were submitted to the GeneBank with accession No. of MH746728.
